# Bibliographical

**Published:** 1873-02

**Authors:** 


### BIBLIOGRAPHICAL.

*Clinical Lectures on Diseases Peculiar to Women.* By Lombe Atthill, M.D., University Dublin; Fellow and Examiner in Midwifery King and Queen's College of Physicians; Vice-President Dublin Obstetrical Society, etc. Second Edition, revised and enlarged, with six lithograph plates and wood-cut illustrations. Philadelphia: Lindsay & Blakiston, Publishers.

This is a careful revision by the author of the edition of 1871—the first edition having been exhausted in less than a year. While, in the main, the author adheres to the views contained in the first edition, some omissions have been supplied and additions made.

This book contains a large amount of clinical experience in a small compass, and is altogether a valuable addition to gynæcological literature. We have been specially interested in his observations upon sub-involution, and for these alone we would commend the work to those who have no large experience in treating diseases of women.

*The Pathology, Diagnosis and Treatment of Diseases of Women, including the Diagnosis of Pregnancy.* By Graily Hewitt, M.D., London, F. R. C. P., Professor of Midwifery and Diseases of Women, University College; Vice-President of the Obstetrical Society of London. Second American from the third London edition. Revised and enlarged, with 132 Illustrations. Philadelphia: Lindsay & Blakiston, Publishers.

The edition before us of this treatise on diseases of women is to a great extent a new work. The first edition only professed to be a summary and criticism of the then existing knowledge on the subject of the diseases of women. The next contained, in addition to the numerous pictorial illustrations of the author's own observations, certain suggestions as to what he regarded as an improved system of uterine pathology. The present work is an elaborate and comprehensive one, containing a more general discussion of the pathology of the uterus—a record of rare and interesting cases and treatment from the author's own experience.

Address all communications regarding in any way the business department of the Journal—viz: those containing money and post-office orders, or relating to advertisements, subscriptions, or failures to receive the Journal—to the publishers, "Herald Publishing Company."

